# Supplementary material for: Maternal anxiety affects embryo implantation via impairing adrenergic receptor signaling in decidual cells
Source: Commun Biol. 2022 Aug 18;5:840. doi: 10.1038/s42003-022-03694-1 (PMC9388523; doi:10.1038/s42003-022-03694-1)

Supplementary Table 1

Table 1 Basal information for control group(n=35) and infertility group(n=35)			
**Variable**	**control group**	**infertility group**	***P***
Average ages(years)of the patients	31.2±4.6	30.9±4.3	*0.782*
Average infertility duration(years)	/	5.5±2.9	*0*
BMI	22.4±3.9	21.5±2.6	*0.425*
Tobacco smoking	2.86%	8.57%	*0.614*
Alcohol consumption	5.70%	0%	*0.493*

BMI, body mass index; values are shown as the mean± standard. *t* tests and χ^2 tests were used to analyze the differences in parameters.

Supplementary Table 2

Table 1 Basal information for pregnancy group(n=43) and pregnant failure group(n=26)			
**Variable**	**pregnancy group**	**RIF**	***P***
Average ages(years)of the patients	30.5±3.6	31.3±2.4	*0.734*
Average infertility duration(years)	4.5±2.6	5.3±1.9	*0.642*
BMI	22.6±5.3	23.2±5.7	*0.523*
Endometrial thickness on the day of embryo transfer	10.3±2.4	8.9±2.8	*0.715*
Alcohol consumption	9.30%	11.54%	*0.533*
Tobacco smoking	4.60%	7.69%	*0.629*
Number of oocytes retrieved	9.9±5.0	9.4±4.5	*0.898*
MIIoocyte rate(n)	86.6 %(393/454)	84.71%(231/275)	*0.198*
Fertilization rate(n)	83.5 %(328/393)	80.1 %(185/231)	*0.169*
2PN rate(n)	74.0 %(291/393)	72.3 %(167/231)	*0.349*
High-quality embryo rate(n)	52.2 %(152/291)	50.7 %(71/140)	*0.423*
Embryo transfer number(D5)	1.21（52/43）	1.38（36/26）	*0.534*

2PN, two pro-nucleate; HCG, human chorionic gonadotropin; D5, Embryos on day 5; values are shown as the mean± standard. *t* tests and χ^2 tests were used to analyze the differences in parameters.

Supplementary Table 3

Table 2 Basal patients information for human endometrial biopsy			
**Variable**	**control (n=6)**	**RIF (n=7)**	***P***
Average ages(years)of the patients	28.7±2.9	30.7±4.6	*0.373*
BMI	21.4±2.7	20.8±2.3	*0.681*
Alcohol consumption	0%	0%	*/*
Tobacco smoking	0%	0%	*/*
Endometrial thickness in Mid-secretory stage	9.3±1.1	9.0±1.5	*0.707*
Basic FSH level (mIU/ml)	5.15±1.1	5.4±0.7	*0.673*
Basic LH level (mIU/ml)	2.16±0.42	2.50±0.85	*0.363*
Basic E2 level (pg/ml)	31.57±5.8	29.67±6.7	*0.595*
The level of LH peak (mIU/ml)	35.0±5.1	33.67±7.7	*0.717*

FSH, Follicle-stimulating hormone; E2, estradiol; LH, Luteinizing hormone; values are shown as the mean± standard. *t* tests and χ^2 tests were used to analyze the differences in parameters.

Supplementary Table 4

Table 4. Quantitative real-time PCR primer sequences

Genes		Primer sequence5’–3’	GenBank accession no.
ADRA1A	Forward	TTCTGCAACATCTGGGCGG	NM_000680.4
	Reverse	GCTGCCTCCAGCCGAACA	
ADRA1B	Forward	GACCTGCTGTTGAGCTTCA	NM_000679.4
	Reverse	AGAGGCCCGATGGAGATGA	
ADRA1D	Forward	GTCACCAACTATTTCATCG	NM_000678.4
	Reverse	GAGATGGTGCAGAGGCT	
ADRA2A	Forward	GAGGAAGAGGAGGACCCA	NM_000681.4
	Reverse	TCGGTCCCGTTCCAGCT	
ADRA2C	Forward	CAGAACCTCTTCCTGGTGT	NM_000683.4
	Reverse	TGCGTCACCGACCAGTAGC	
ADRA2B	Forward	CTGGCCAACGAGCTGCTG	NM_000682.7
	Reverse	GTGCGCTTGGAGTTGTACT	
ADRB1	Forward	GTGCTGGTGATCGTGGCCAT	NM_000684.3
	Reverse	GCAGAAGAAGGAGCCGTACTC	
ADRB2	Forward	ATCGTCCTGGCCATCGTG	NM_000024.5
	Reverse	ATCACGCACAGGGTCTC	
ADRB3	Forward	ACCAACGTGTTCGTGACTTC	NM_000025.3
	Reverse	CACCACTGGCTCATGATGG	
PNMT	Forward	CTCCGCAACAACTACGCG	NM_002686.4
	Reverse	TCTGTCATGGTGATGTCC	
TH	Forward	GACTGCTGCCACGAGCTG	XM_011520335.2
	Reverse	TCTTGGTAGGGCTGCAC	
GAPDH	Forward	ATTTGGCTACAGCAACAGG	NM_002046.7
	Reverse	TTGAGCACAGGGTACTTTATT	

Supplementary Fig. S1

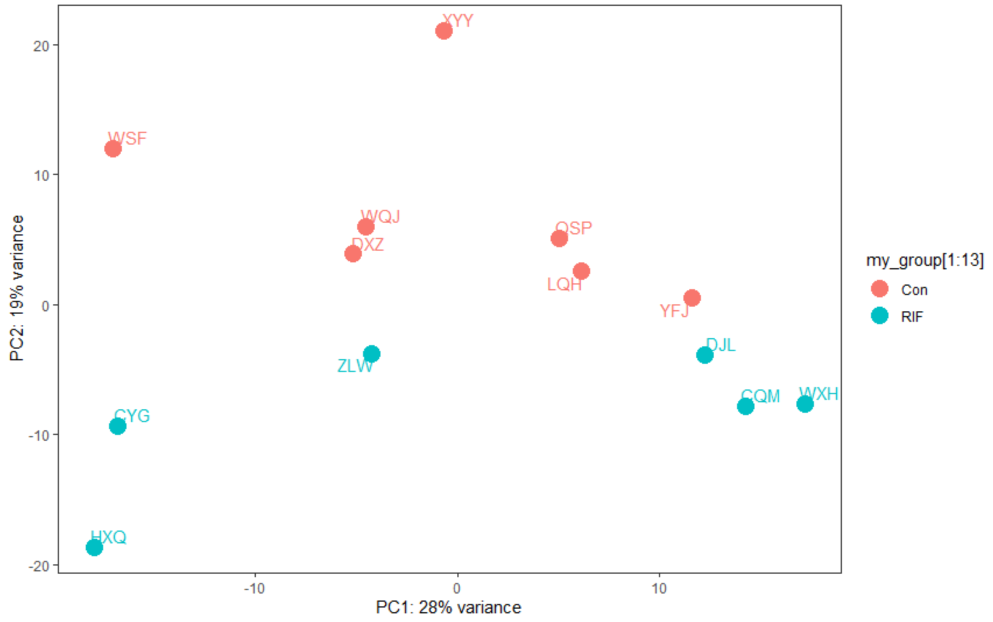

Supplementary Fig. S2

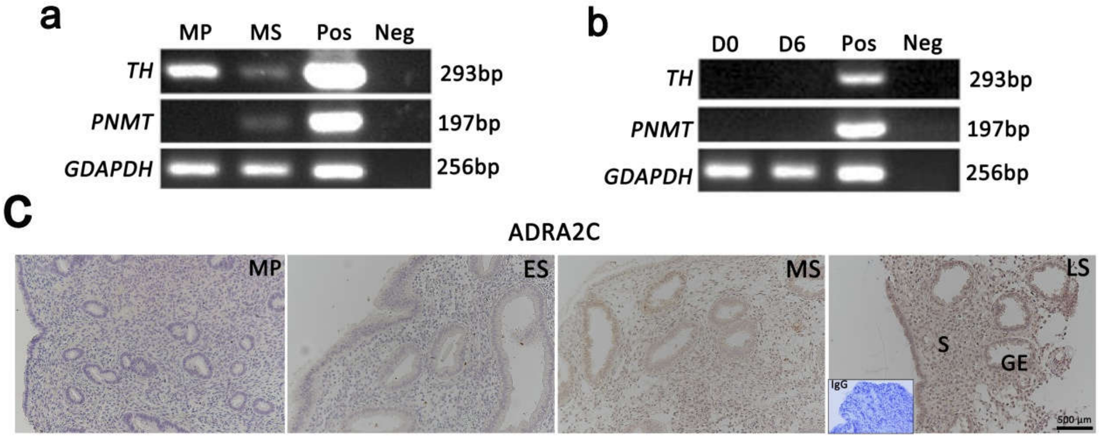

a The expression of *TH* and *PNMT* in endometrial at different menstrual stages; **b** The expression of *TH* and *PNMT* before and after HESCs differentiation; **c** Immunohistochemistry showed ADRA2C protein expression in endometrium from proliferative stage to late secretory phase of the menstrual cycle. Scale bars, 500 μM. Pos, positive; Neg, negative; MP, Mid-proliferationphase; MS, Mid -secretion phase; ES, ES, early secretory phase; LS, late secretory phase; S, stroma; GE, glandular epithelium; IgG for control;

Supplementary Fig. S3: **Effects of adrenergic receptor signaling on proliferation of HESCs during in vitro culture.**

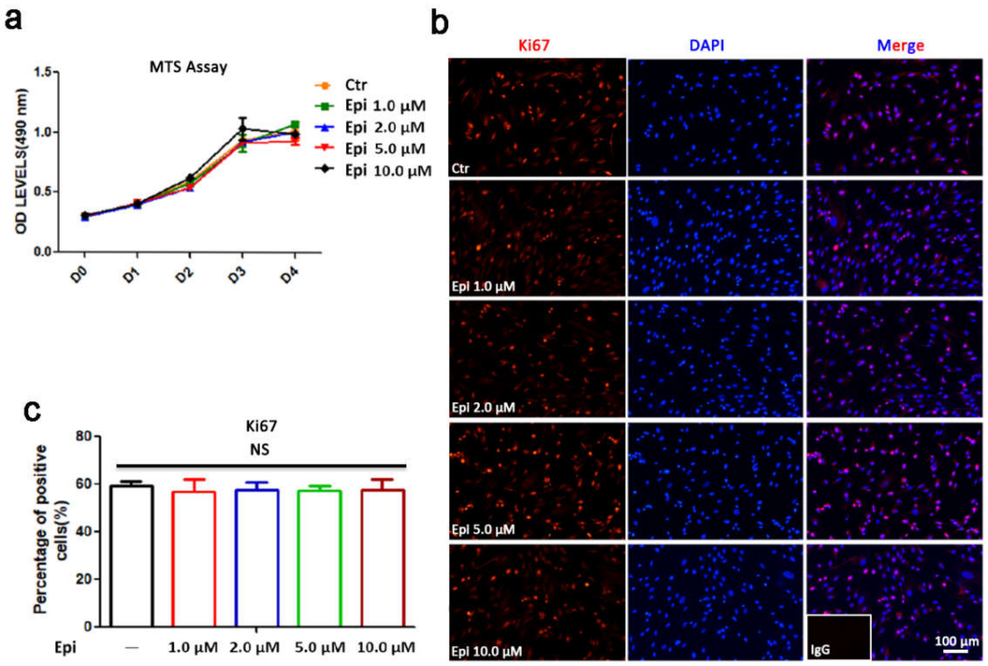

a MTS assay monitored the proliferation of human endometrial stromal cells in vitroculture; **b** Ki67 immunofluorescence assay analyzed the proliferation of human endometrial stromal cells in vitroculture; **c** The positive rate of Ki67 positive cells; Ctr, control; NS, No significance. IgG for control;

Supplementary Fig. S4

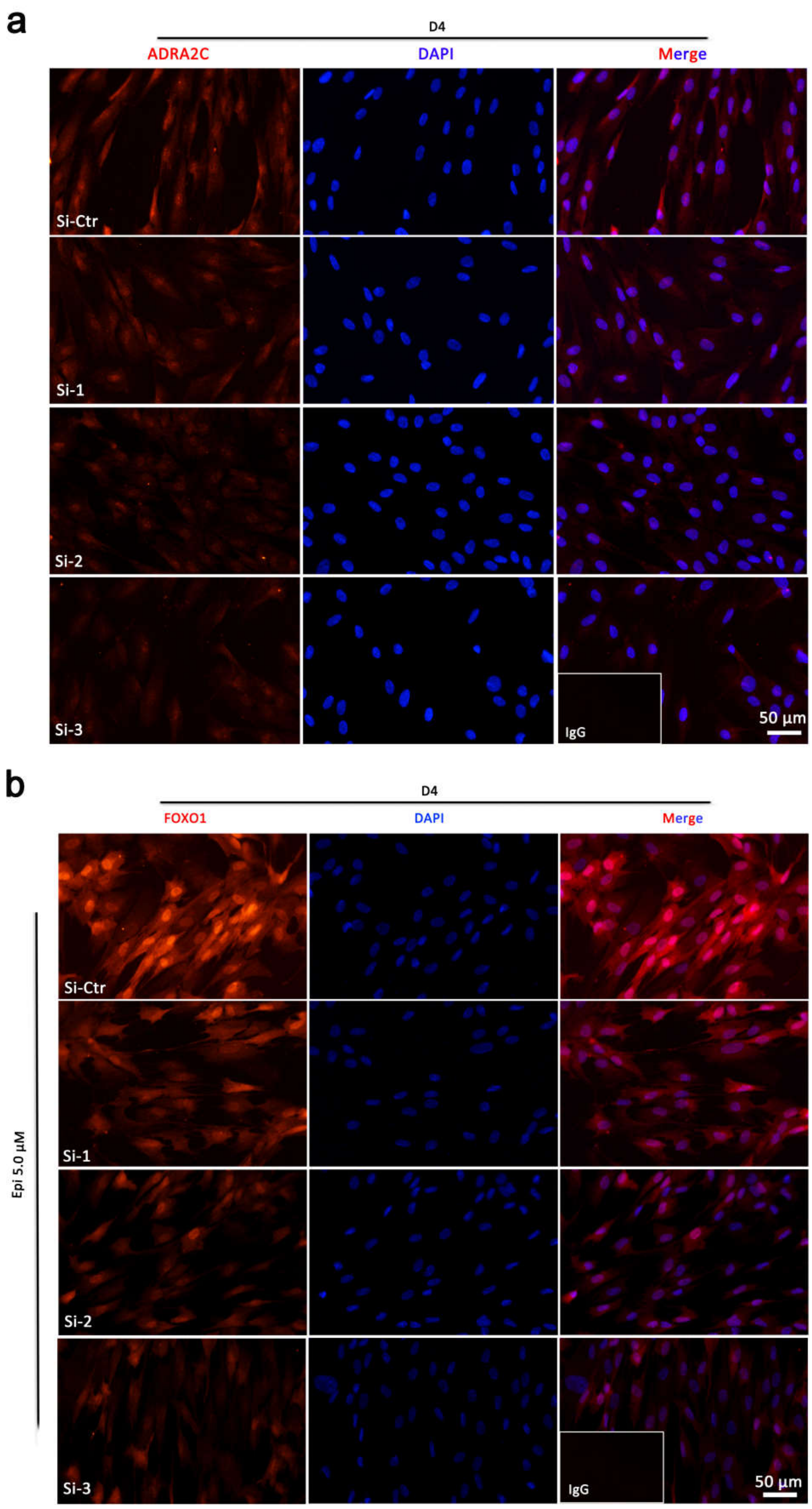

a Immunofluorescence detected the knockdown efficiency of ADRA2C in HESCs; **b** Expression and localization of FOXO1 by cellular immunofluorescence assay after ADRA2C knockdown; Scale bars, 50 μM

Supplementary Figure S5

PCR screen for adrenergic receptors

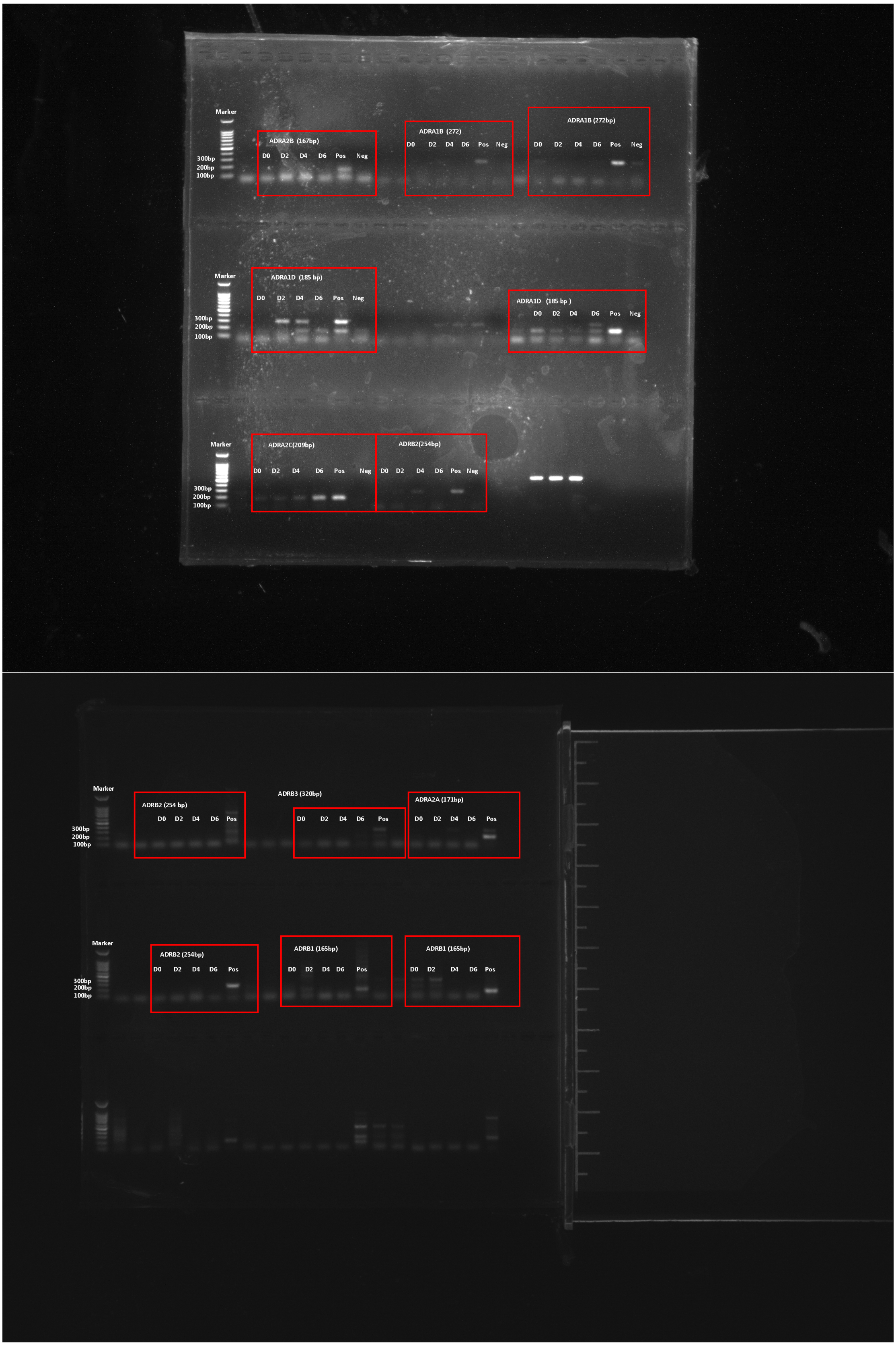

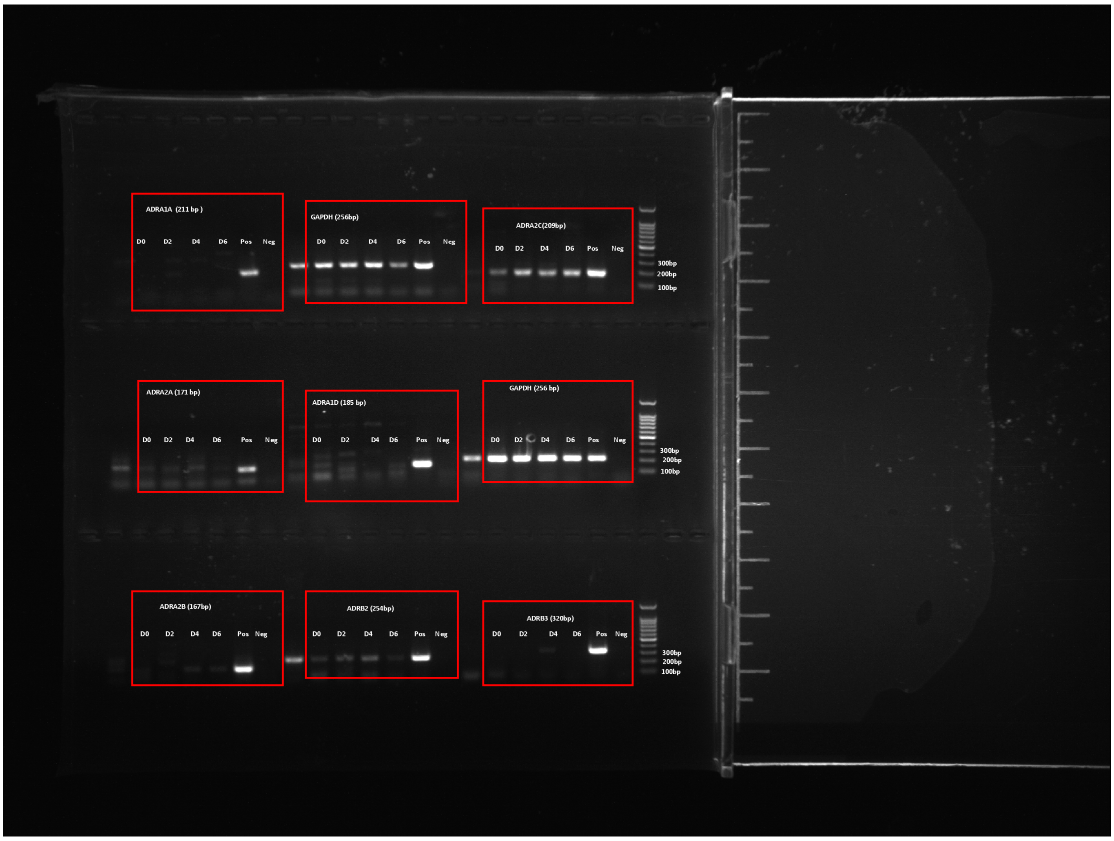

Figure 2 unedited blots

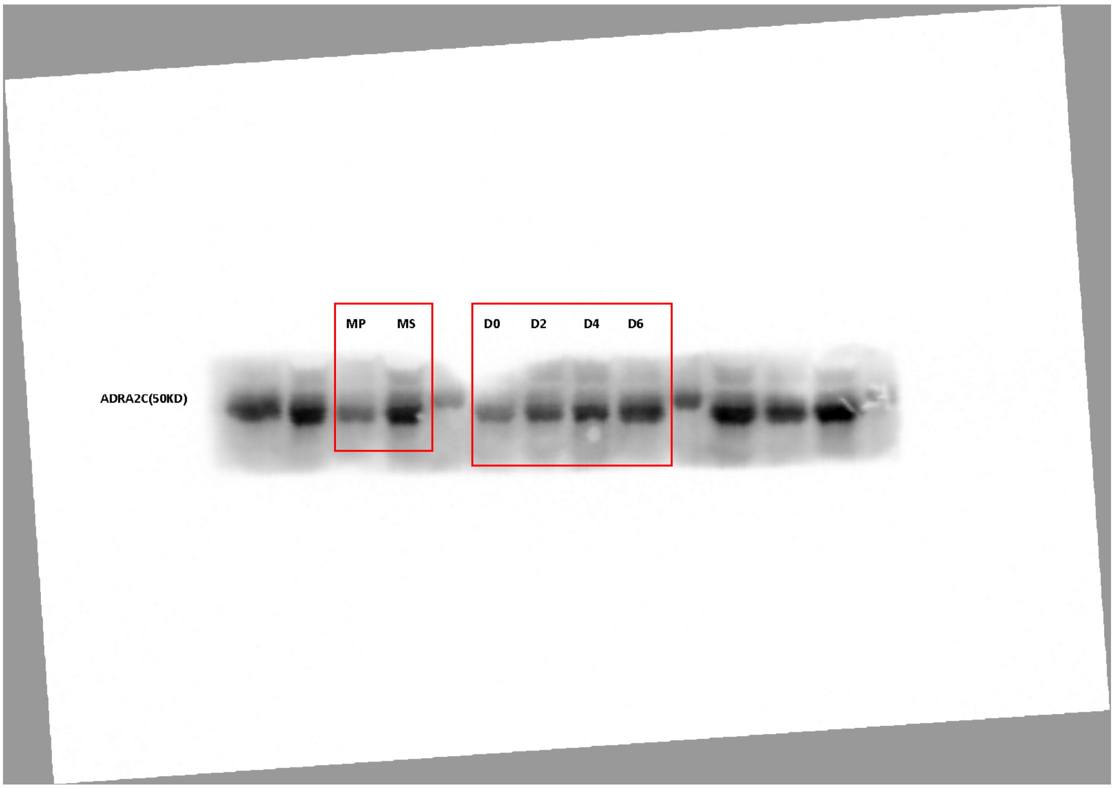

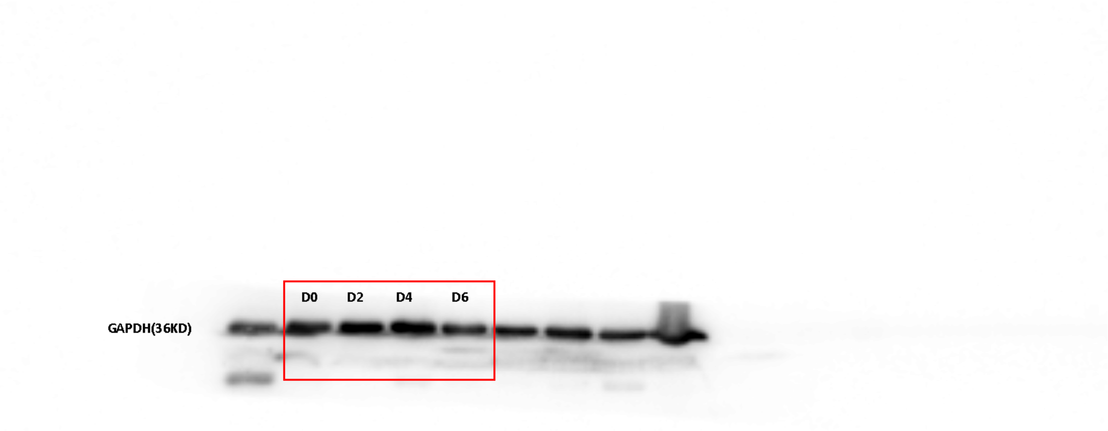
GAPDH(36KD)
D0
D2
D4
D6

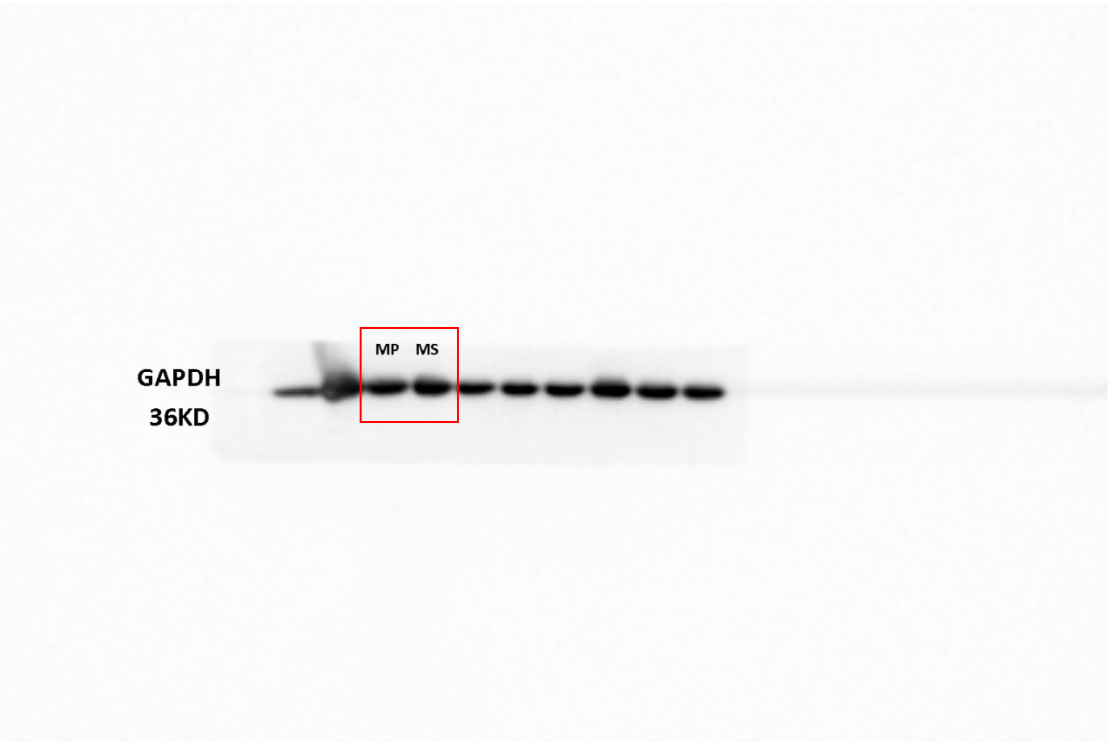
GAPDH
36KD
MP
MS

Figure 3 unedited blots

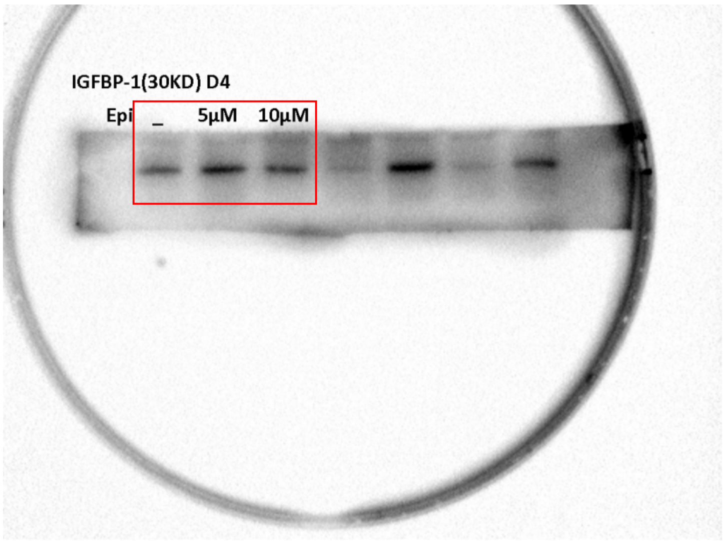

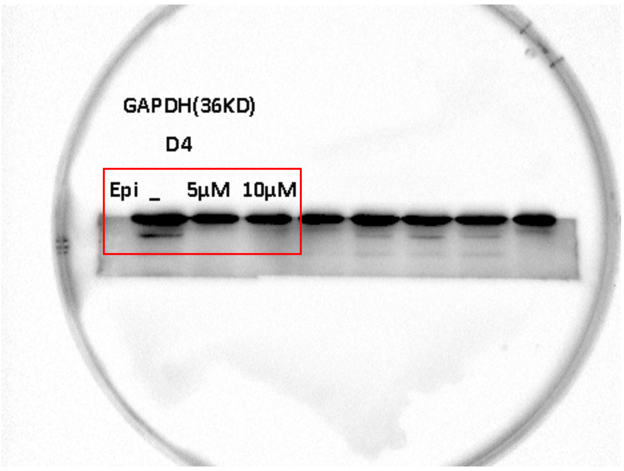

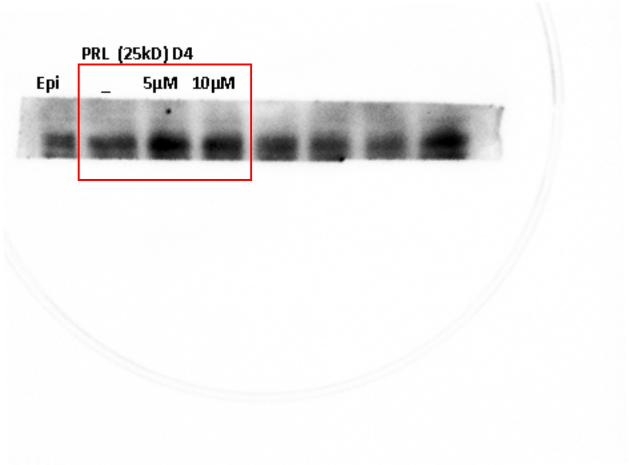

Figure 4a unedited blots

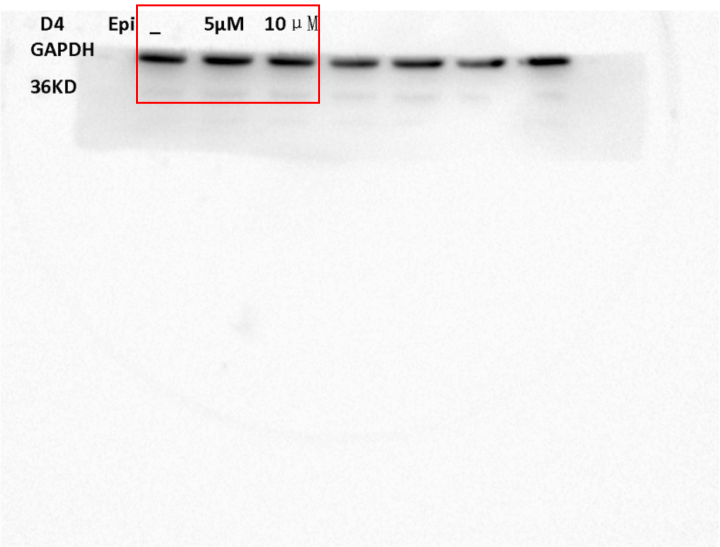

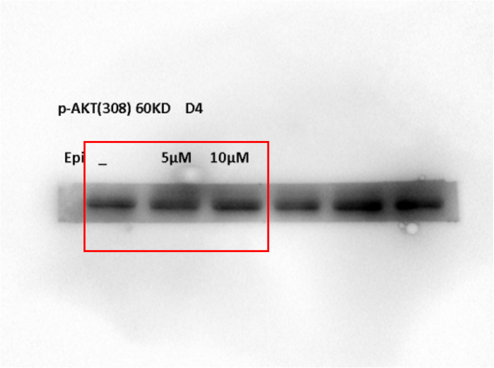

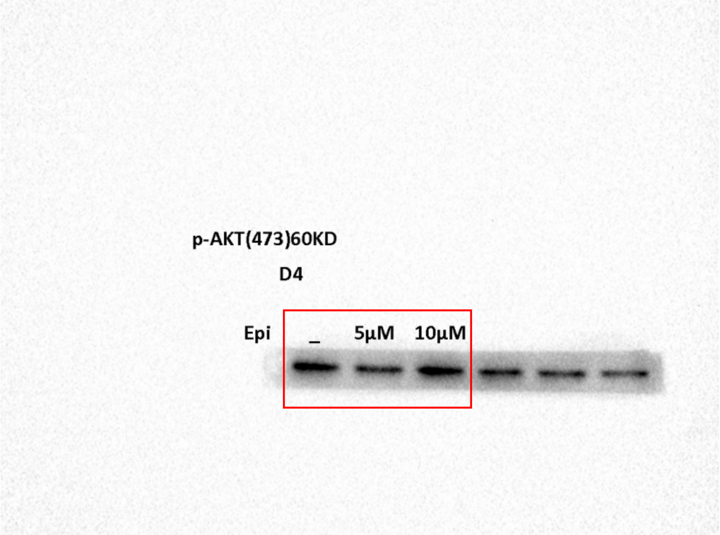

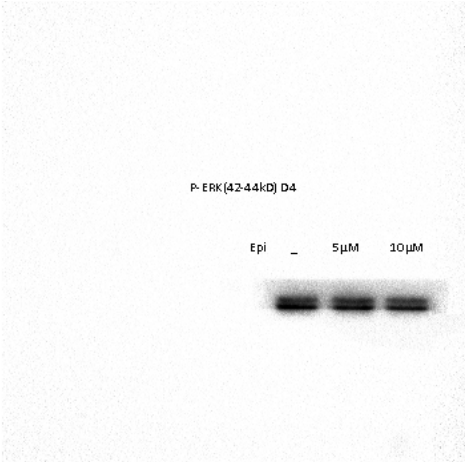
P- ERK(42-44kD) D4
Epi
_
5μM
10μM

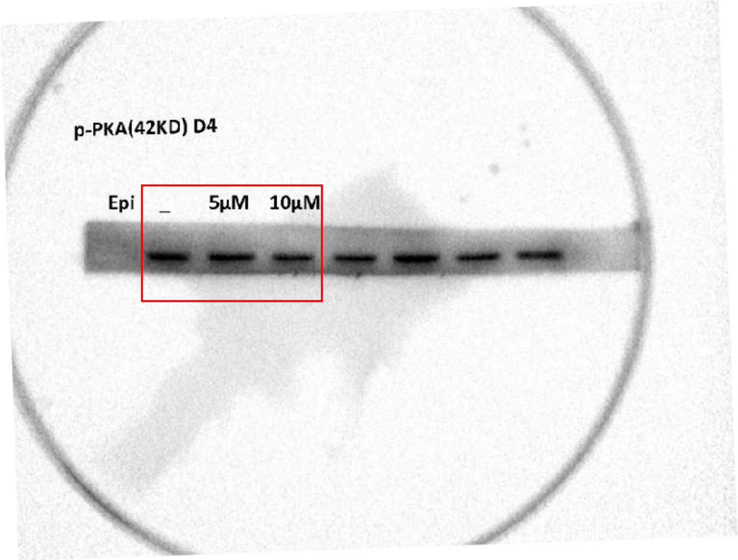
p-PKA(42KD) D4
Epi
_
5μM
10μM

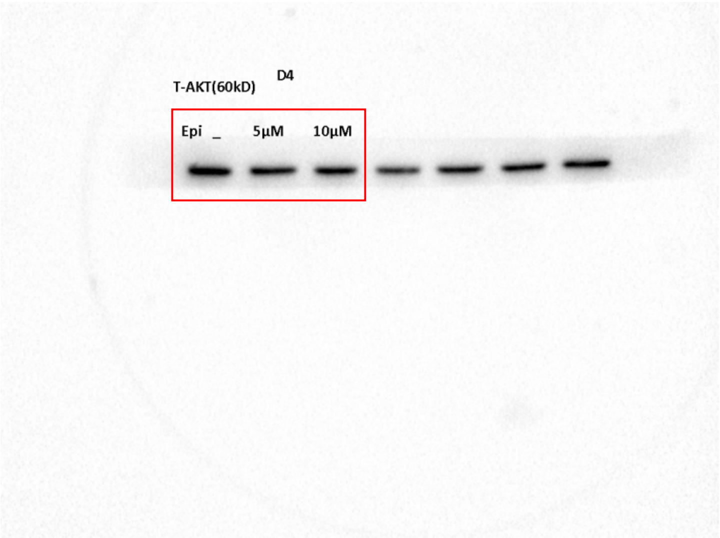
T-AKT(60kD)
D4
Epi
_
5μM
10μM

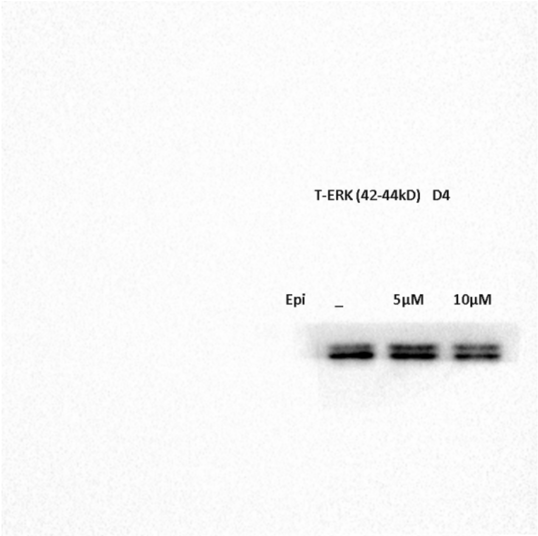

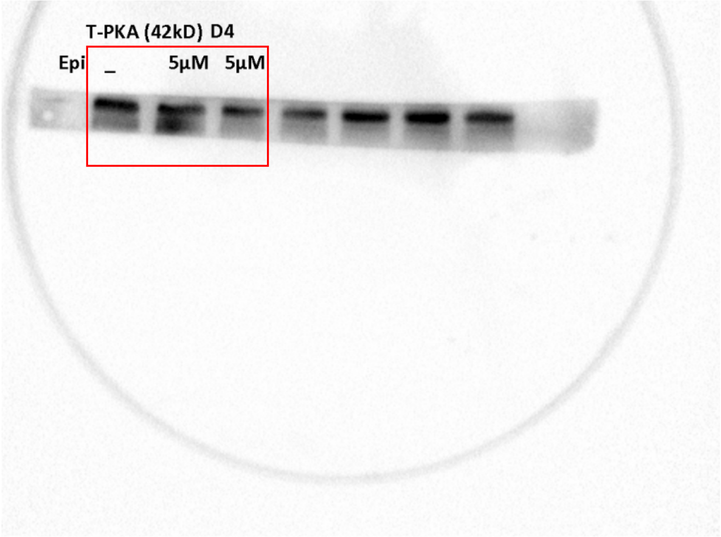

Figure 4c unedited blots

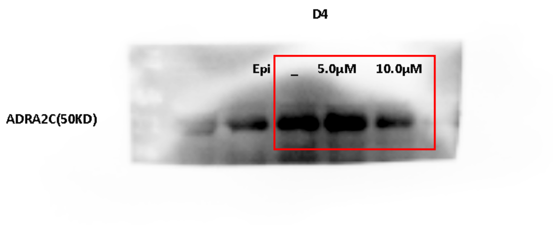
D4
Epi
_
5.0μM
10.0μM
ADRA2C(50KD)

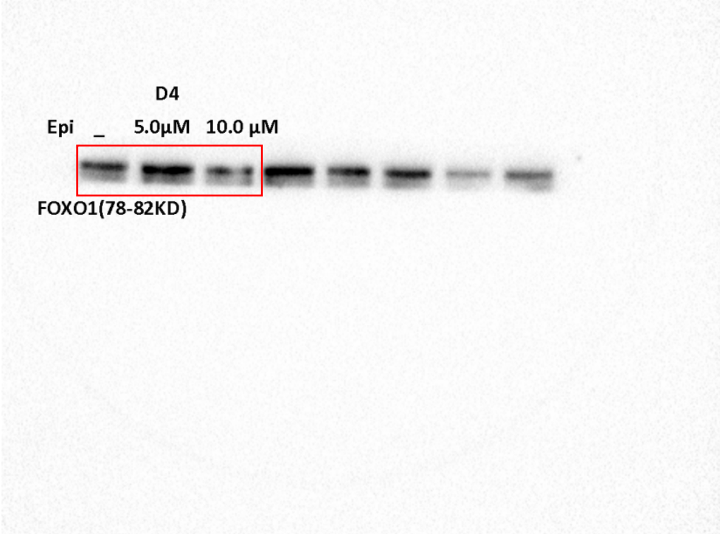
D4
Epi
_
5.0μM
10.0 μM
FOXO1(78-82KD)

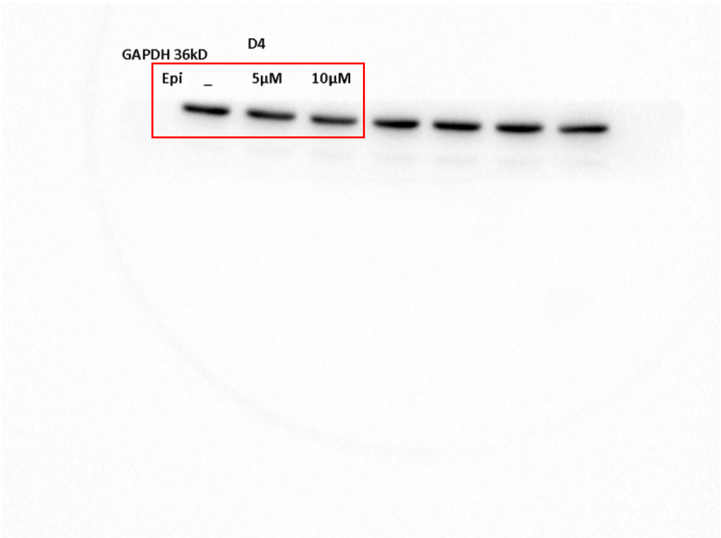
GAPDH 36kD
D4
Epi
_
5μM
10μM

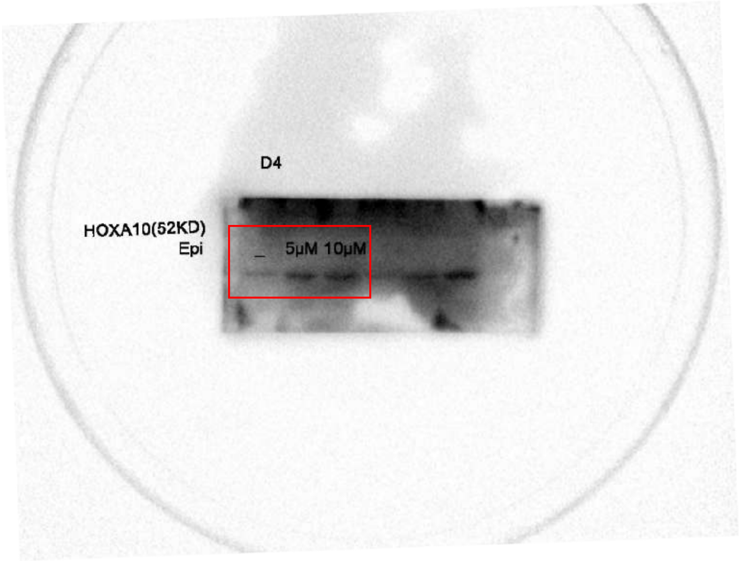

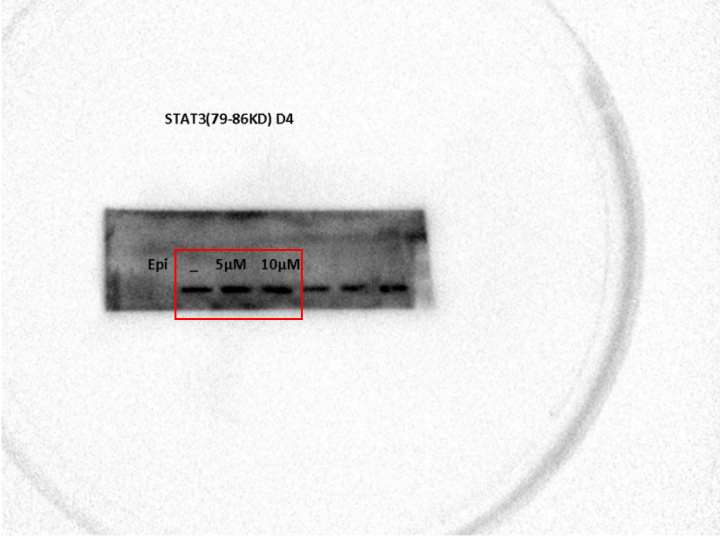

Figure 4e unedited blots

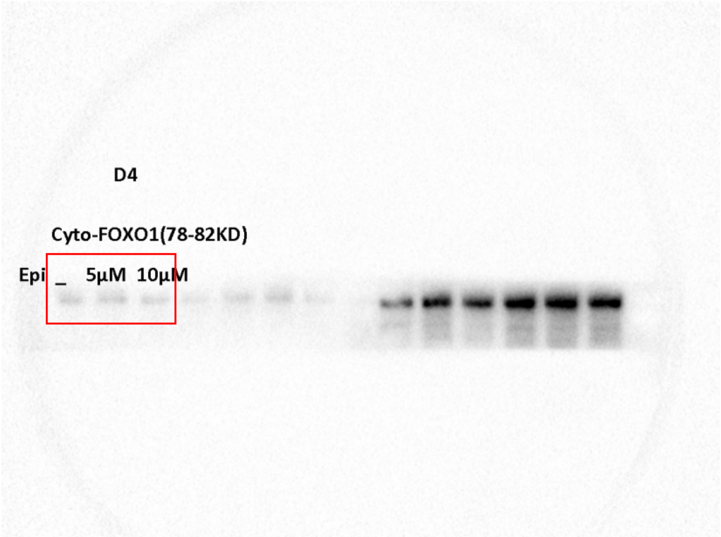

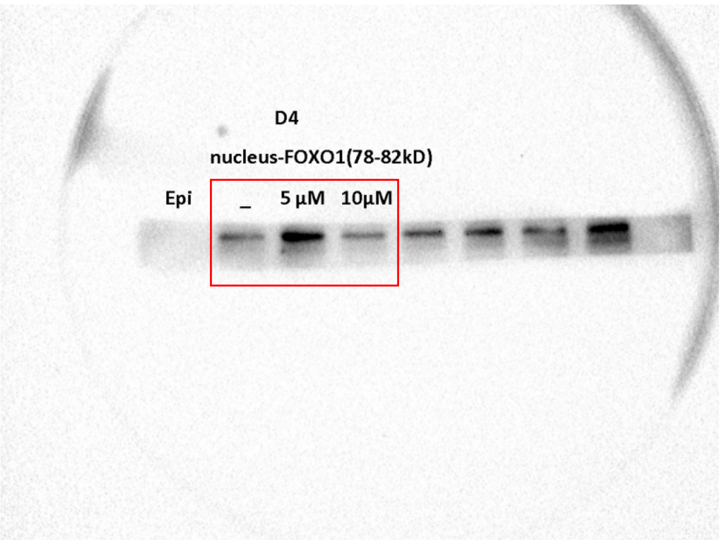
D4
nucleus-FOXO1(78-82kD)
Epi
_
5 μM
10μM

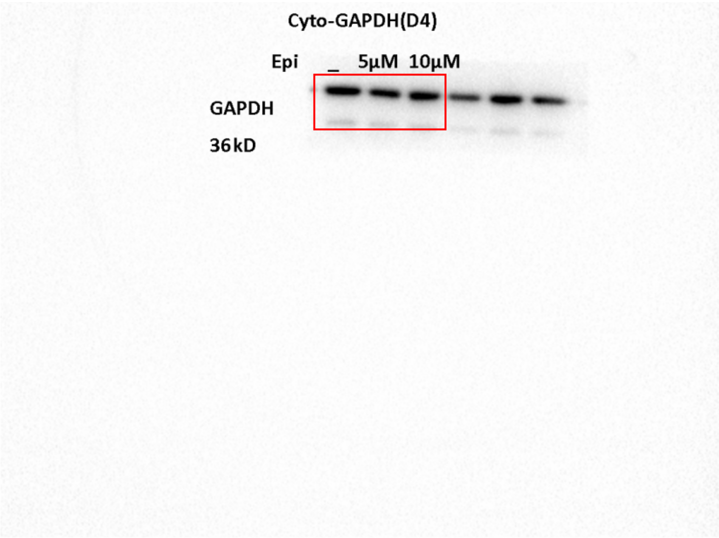
Cyto-GAPDH(D4)
Epi
_
5μM
10μM
GAPDH
36kD

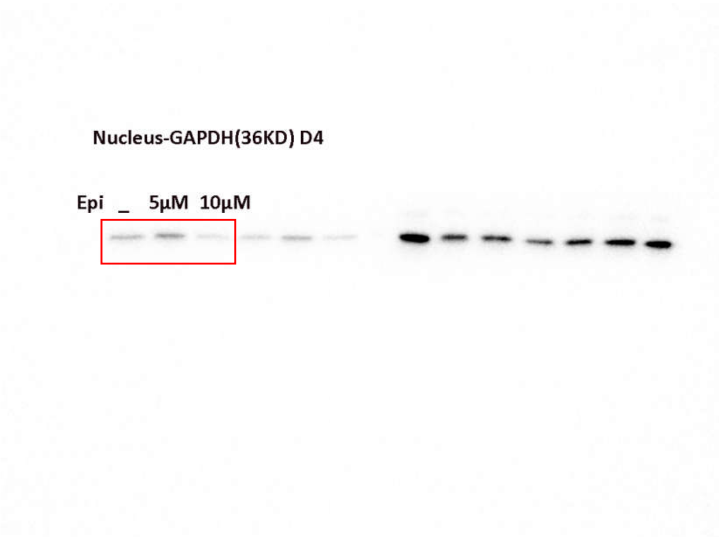
Nucleus-GAPDH(36KD) D4
Epi
_
5μM
10μM

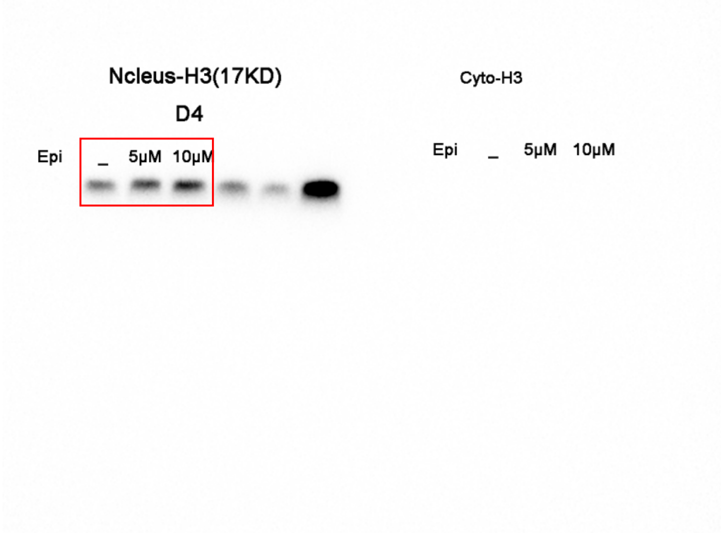

Figure 5b unedited blots

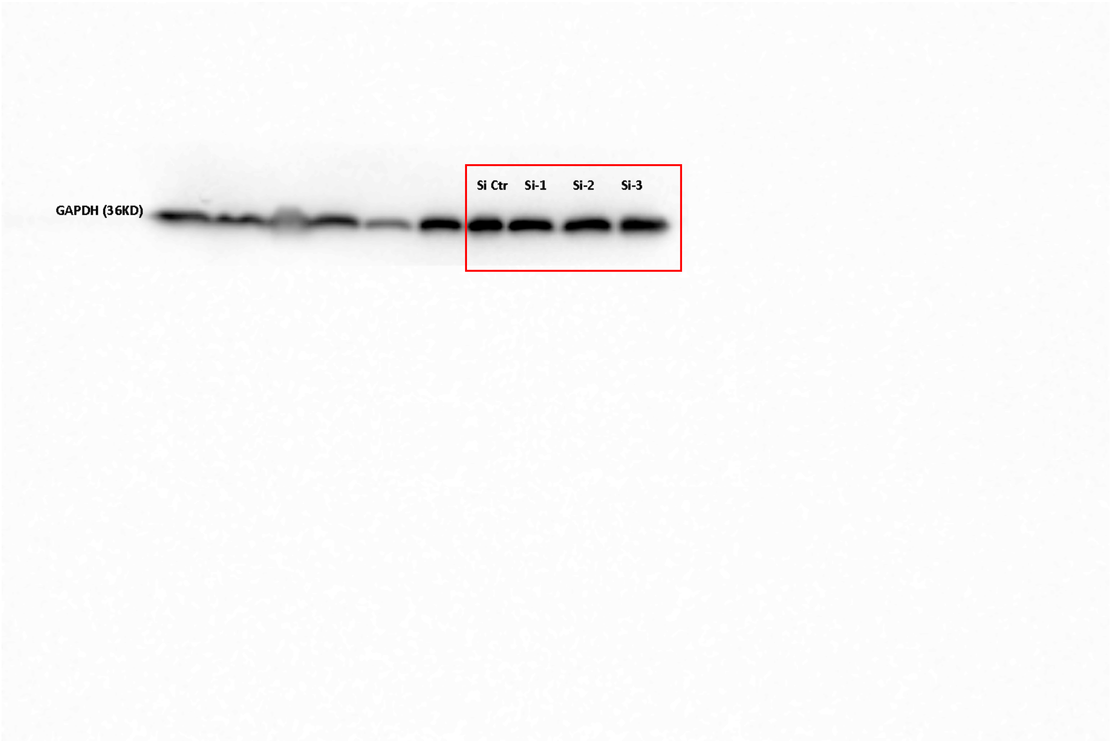

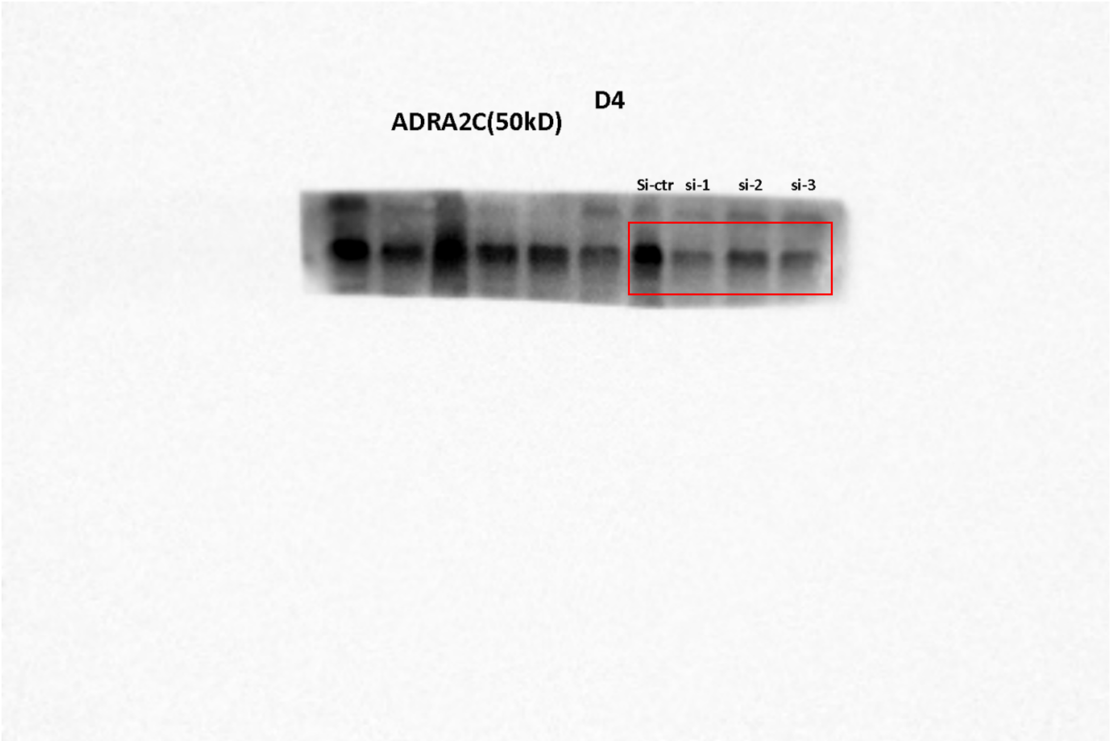

Figure 5f unedited blots

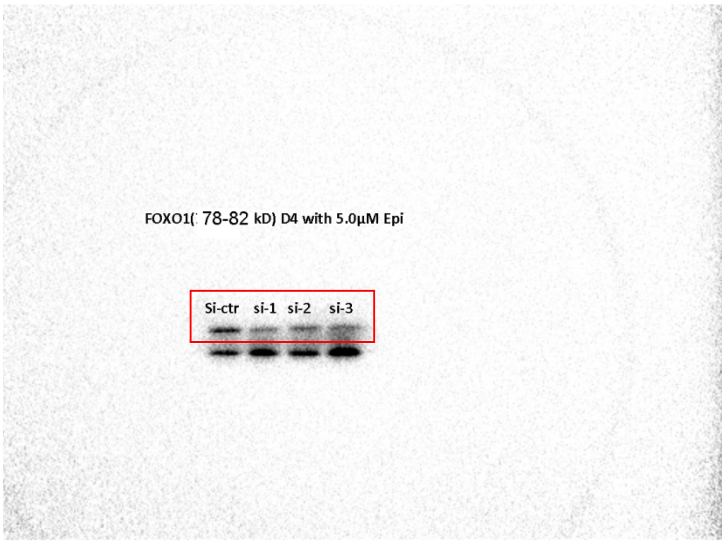

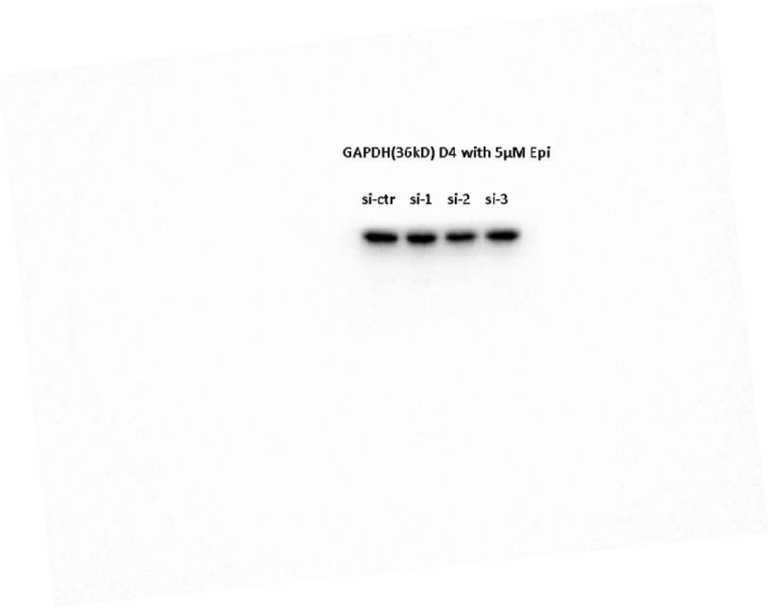
GAPDH(36kD) D4 with 5μM Epi
si-ctr si-1 si-2 si-3

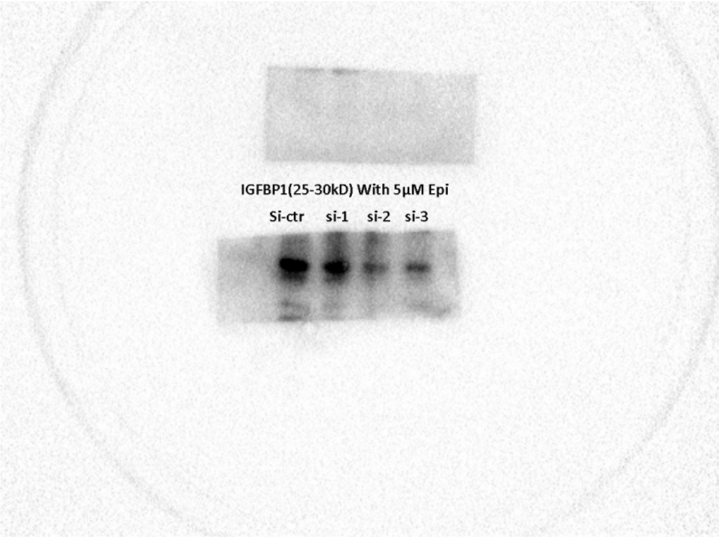
IGFBP1(25-30kD) With 5μM Epi
Si-ctr si-1 si-2 si-3

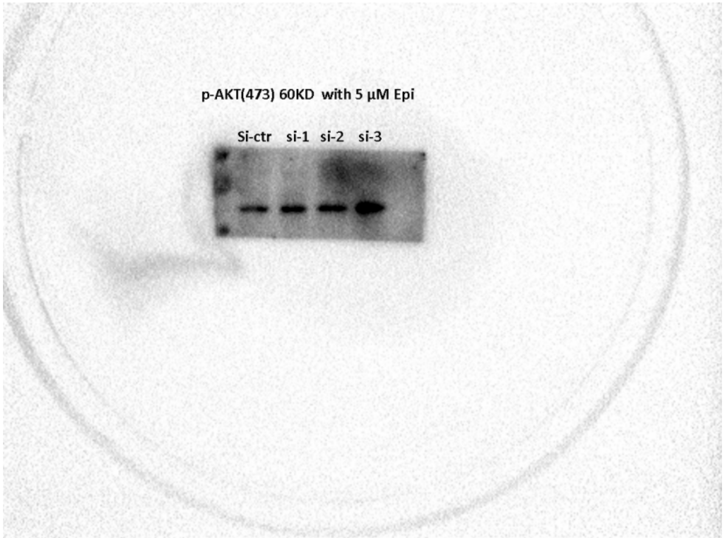
p-AKT(473) 60KD with 5 μM Epi
Si-ctr si-1 si-2 si-3

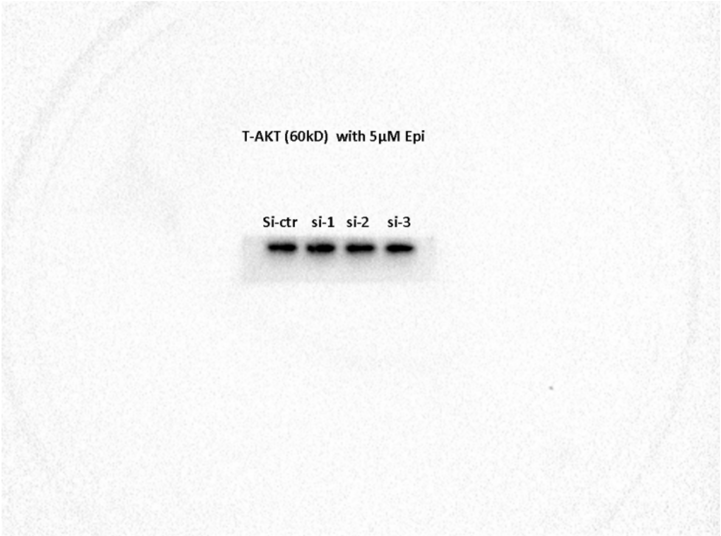

Figure 5h unedited blots

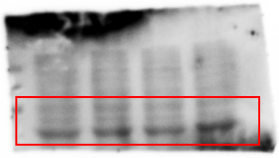

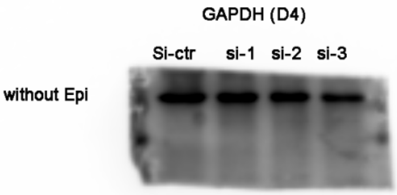
GAPDH (D4)
Si-ctr
si-1
si-2
si-3
without Epi

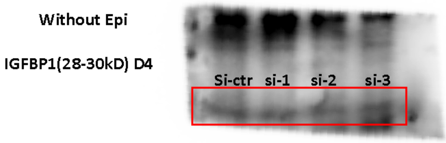
Without Epi
IGFBP1(28-30kD) D4
Si-ctr
si-1
si-2
si-3

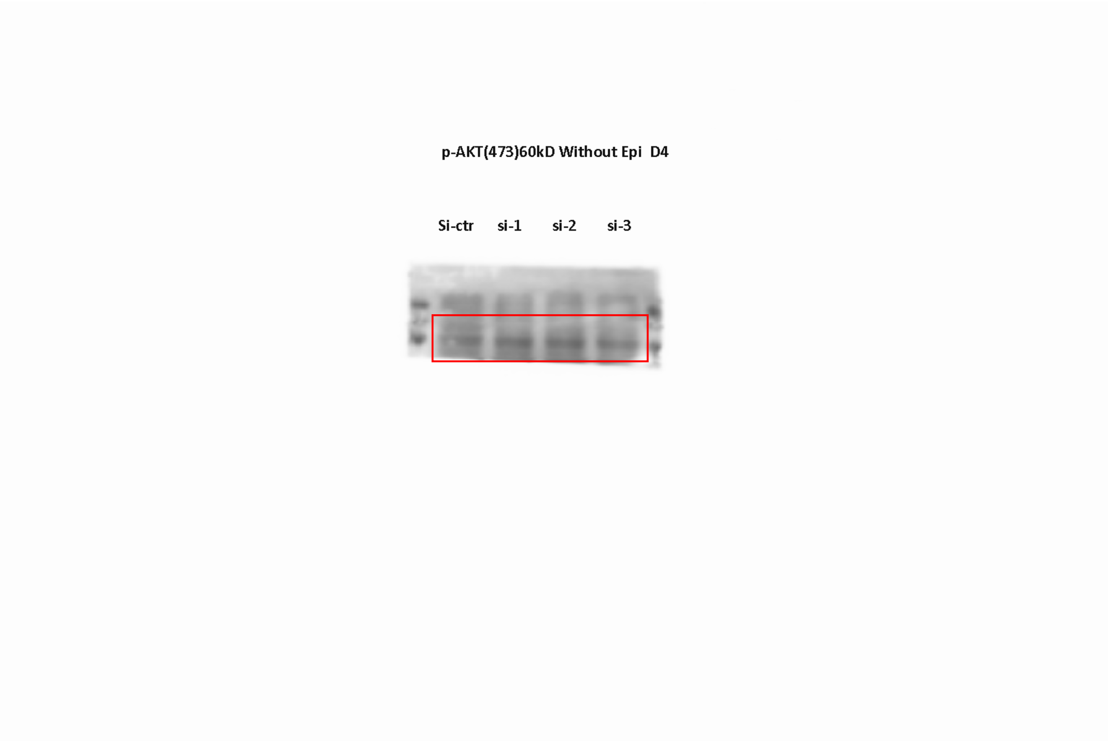

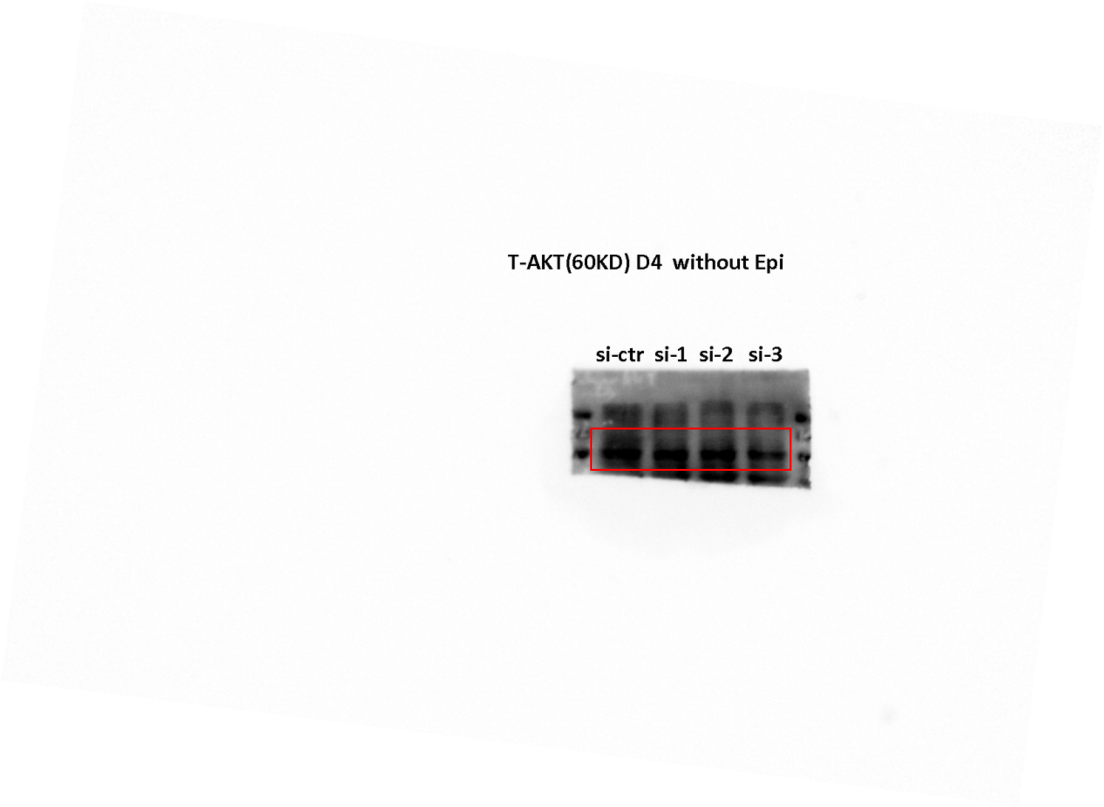

Figure 5j unedited blots

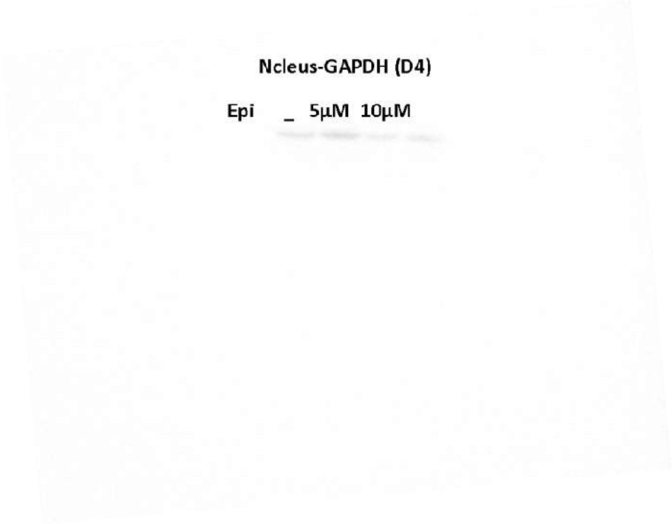
Ncleus-GAPDH (D4)
Epi _ 5μM 10μM

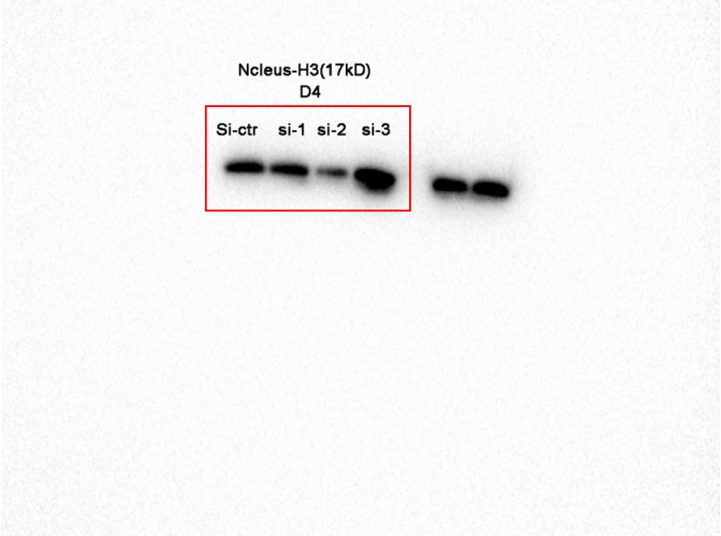
Ncleus-H3(17kD)
D4
Si-ctr si-1 si-2 si-3

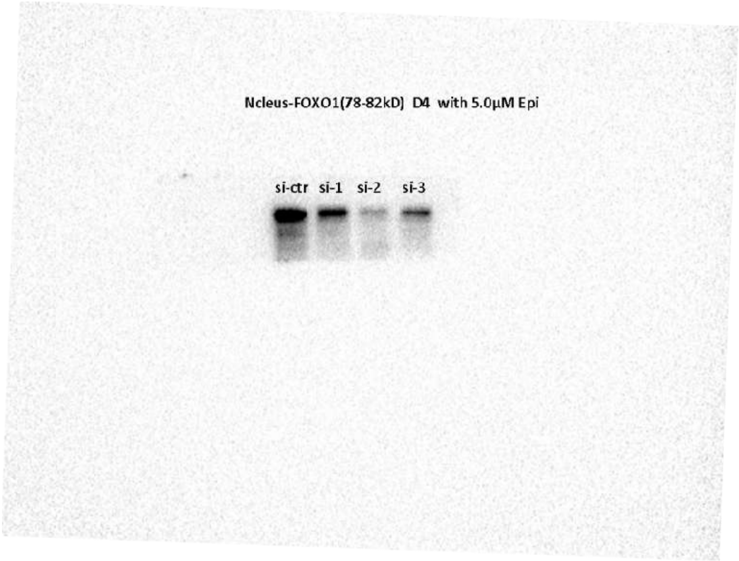
Ncleus-FOXO1(78-82kD) D4 with 5.0μM Epi
si-ctr si-1 si-2 si-3

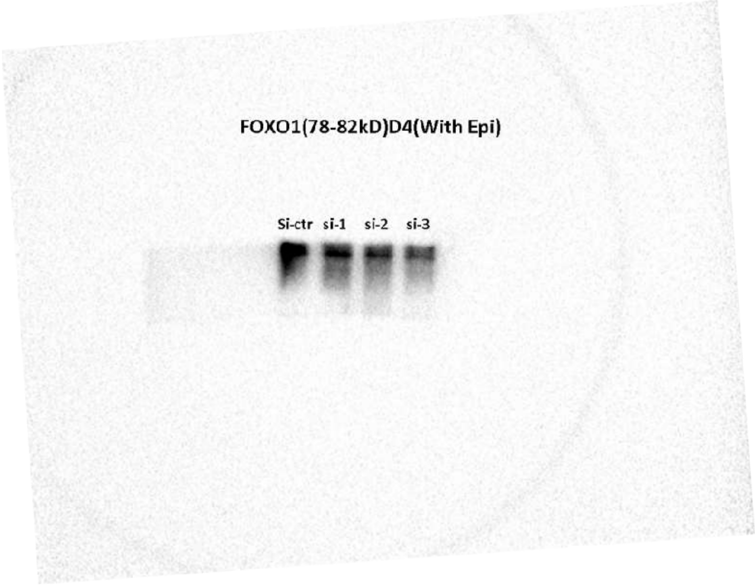
FOXO1(78-82kD)D4(With Epi)
Si-ctr si-1 si-2 si-3

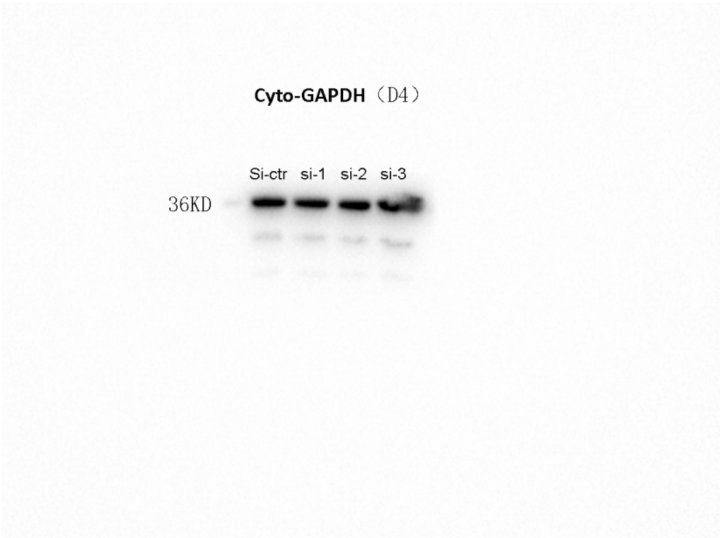
Cyto-GAPDH（D4）
Si-ctr si-1 si-2 si-3
36KD

Figure 7h unedited blots

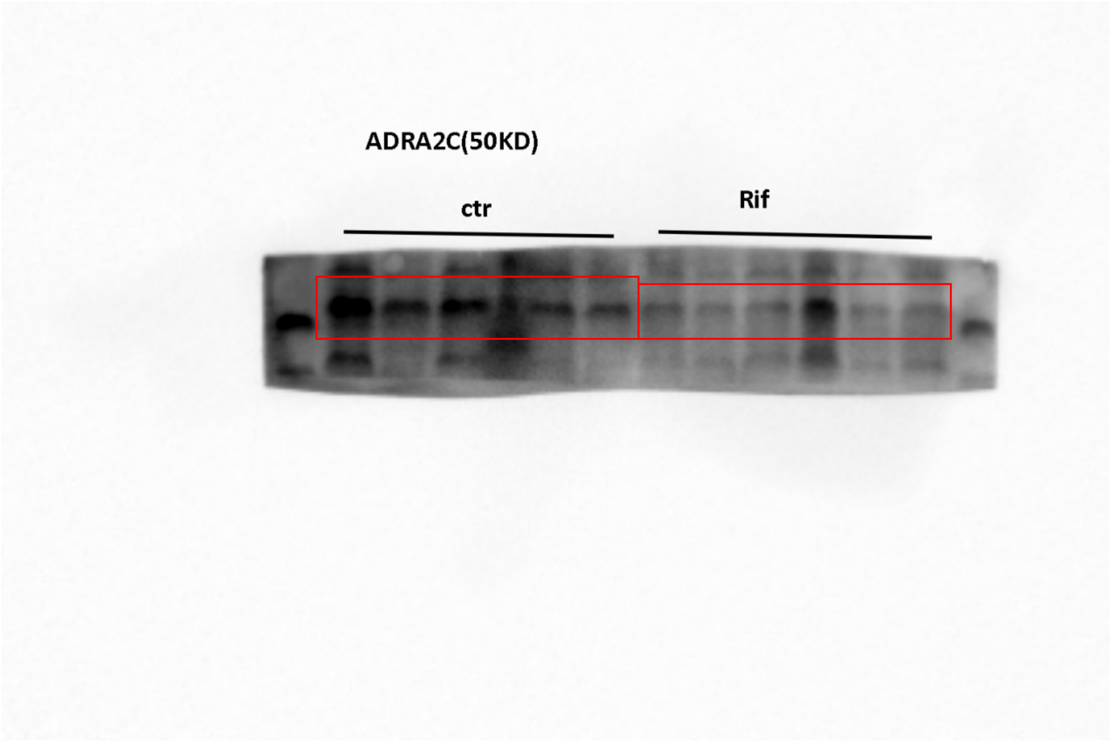

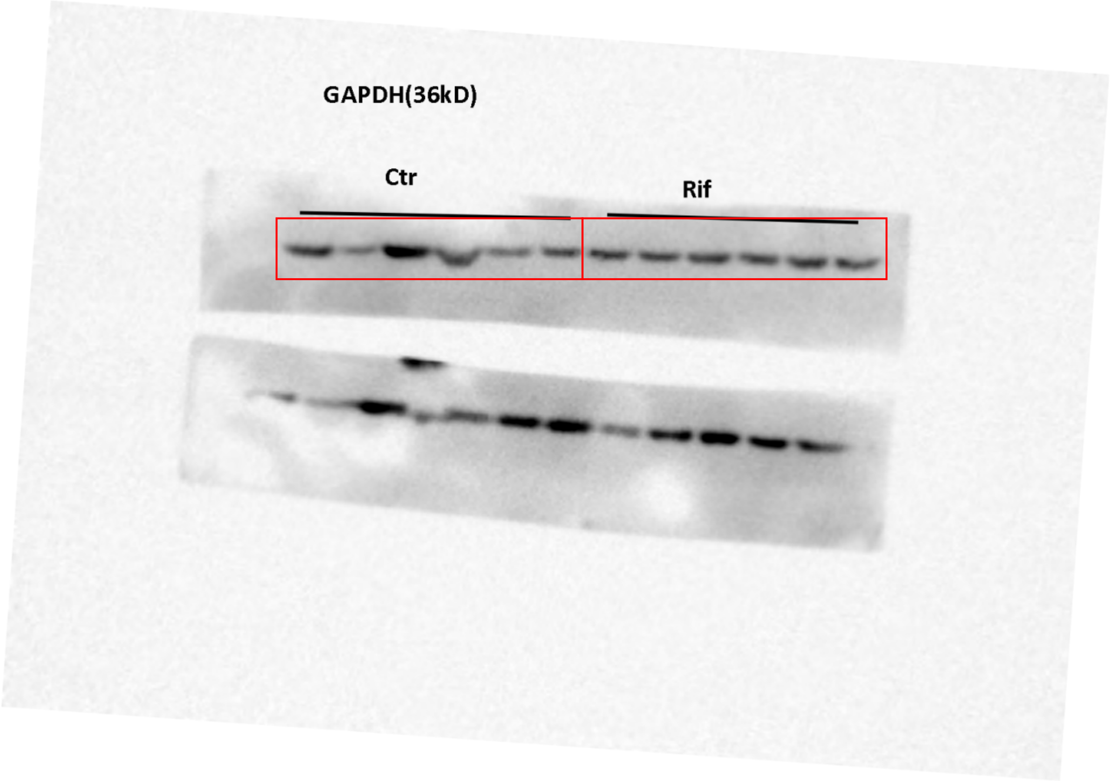

Supplement: Supplementary file 2 — Supplementary Information [file 42003_2022_3694_MOESM2_ESM.pdf]
