# Supplementary material for: Maternal anxiety affects embryo implantation via impairing adrenergic receptor signaling in decidual cells
Source: Commun Biol. 2022 Aug 18;5:840. doi: 10.1038/s42003-022-03694-1 (PMC9388523; doi:10.1038/s42003-022-03694-1)
Supplement: Supplementary file 3 — Description of Additional Supplementary Files [file 42003_2022_3694_MOESM3_ESM.pdf]

Description of Additional Supplementary Files

File name: Supplementary Data
Description: All source data for graphs and charts displayed in the main chart.
