# Supplementary material for: Maternal anxiety affects embryo implantation via impairing adrenergic receptor signaling in decidual cells
Source: Commun Biol. 2022 Aug 18;5:840. doi: 10.1038/s42003-022-03694-1 (PMC9388523; doi:10.1038/s42003-022-03694-1)
Supplement: Supplementary file 5 — Reporting Summary [file 42003_2022_3694_MOESM5_ESM.pdf]

nature portfolio

Corresponding author(s): Jinxiang Wu, Shuangbo Kong, Haibin Wang
Last updated by author(s): Jinxiang Wu; Shu Lin; Pinxiu Huang; Lingling

# Reporting Summary

Nature Portfolio wishes to improve the reproducibility of the work that we publish. This form provides structure for consistency and transparency in reporting. For further information on Nature Portfolio policies, see our Editorial Policies and the Editorial Policy Checklist.

## Statistics

For all statistical analyses, confirm that the following items are present in the figure legend, table legend, main text, or Methods section.

| n/a | Confirmed | |
|---|---|---|
| ☐ | ☒ | The exact sample size (*n*) for each experimental group/condition, given as a discrete number and unit of measurement |
| ☐ | ☒ | A statement on whether measurements were taken from distinct samples or whether the same sample was measured repeatedly |
| ☐ | ☒ | The statistical test(s) used AND whether they are one- or two-sided<br>*Only common tests should be described solely by name; describe more complex techniques in the Methods section.* |
| ☐ | ☒ | A description of all covariates tested |
| ☐ | ☒ | A description of any assumptions or corrections, such as tests of normality and adjustment for multiple comparisons |
| ☐ | ☒ | A full description of the statistical parameters including central tendency (e.g. means) or other basic estimates (e.g. regression coefficient) AND variation (e.g. standard deviation) or associated estimates of uncertainty (e.g. confidence intervals) |
| ☐ | ☒ | For null hypothesis testing, the test statistic (e.g. *F*, *t*, *r*) with confidence intervals, effect sizes, degrees of freedom and *P* value noted<br>*Give P values as exact values whenever suitable.* |
| ☒ | ☐ | For Bayesian analysis, information on the choice of priors and Markov chain Monte Carlo settings |
| ☒ | ☐ | For hierarchical and complex designs, identification of the appropriate level for tests and full reporting of outcomes |
| ☒ | ☐ | Estimates of effect sizes (e.g. Cohen's *d*, Pearson's *r*), indicating how they were calculated |

*Our web collection on statistics for biologists contains articles on many of the points above.*

## Software and code

Policy information about availability of computer code

| | |
|---|---|
| Data collection | Image Lab manager software, Bioanalyzer2100 system and Nikon NIS Elements D 4.00.12 software. |
| Data analysis | Image J, GraphPad Prism 5, edgeR package(version 3.26.5), ggplot2 (version 3.2.0), org.Hs.eg.db (version 3.8.2), cluster Profiler packages (version 2.1.1). |

For manuscripts utilizing custom algorithms or software that are central to the research but not yet described in published literature, software must be made available to editors and reviewers. We strongly encourage code deposition in a community repository (e.g. GitHub). See the Nature Portfolio guidelines for submitting code & software for further information.

## Data

Policy information about availability of data

All manuscripts must include a data availability statement. This statement should provide the following information, where applicable:
- Accession codes, unique identifiers, or web links for publicly available datasets
- A description of any restrictions on data availability
- For clinical datasets or third party data, please ensure that the statement adheres to our policy

RNA-seq data generated for this study will be available at NCBI GEO accession .

# Field-specific reporting

Please select the one below that is the best fit for your research. If you are not sure, read the appropriate sections before making your selection.

☒ Life sciences ☐ Behavioural & social sciences ☐ Ecological, evolutionary & environmental sciences

For a reference copy of the document with all sections, see nature.com/documents/nr-reporting-summary-flat.pdf

# Life sciences study design

All studies must disclose on these points even when the disclosure is negative.

| | |
|---|---|
| Sample size | Individual data points from replicate experiments are shown in all figures. All figure legends describe the sample size for individual graphs. For RNA-seq we had n>3 samples for each group which is standard for RNA-seq analysis of the tissues. |
| Data exclusions | No data were excluded from the study. |
| Replication | As mentioned with all relevant figures, sufficient technical and biological replicates of the experiments were performed and the data is reproducible using the methods and models described by the authors. |
| Randomization | N/A |
| Blinding | N/A |

# Behavioural & social sciences study design

All studies must disclose on these points even when the disclosure is negative.

| | |
|---|---|
| Study description | *Briefly describe the study type including whether data are quantitative, qualitative, or mixed-methods (e.g. qualitative cross-sectional, quantitative experimental, mixed-methods case study).* |
| Research sample | *State the research sample (e.g. Harvard university undergraduates, villagers in rural India) and provide relevant demographic information (e.g. age, sex) and indicate whether the sample is representative. Provide a rationale for the study sample chosen. For studies involving existing datasets, please describe the dataset and source.* |
| Sampling strategy | *Describe the sampling procedure (e.g. random, snowball, stratified, convenience). Describe the statistical methods that were used to predetermine sample size OR if no sample-size calculation was performed, describe how sample sizes were chosen and provide a rationale for why these sample sizes are sufficient. For qualitative data, please indicate whether data saturation was considered, and what criteria were used to decide that no further sampling was needed.* |
| Data collection | *Provide details about the data collection procedure, including the instruments or devices used to record the data (e.g. pen and paper, computer, eye tracker, video or audio equipment) whether anyone was present besides the participant(s) and the researcher, and whether the researcher was blind to experimental condition and/or the study hypothesis during data collection.* |
| Timing | *Indicate the start and stop dates of data collection. If there is a gap between collection periods, state the dates for each sample cohort.* |
| Data exclusions | *If no data were excluded from the analyses, state so OR if data were excluded, provide the exact number of exclusions and the rationale behind them, indicating whether exclusion criteria were pre-established.* |
| Non-participation | *State how many participants dropped out/declined participation and the reason(s) given OR provide response rate OR state that no participants dropped out/declined participation.* |
| Randomization | *If participants were not allocated into experimental groups, state so OR describe how participants were allocated to groups, and if allocation was not random, describe how covariates were controlled.* |

# Ecological, evolutionary & environmental sciences study design

All studies must disclose on these points even when the disclosure is negative.

| | |
|---|---|
| Study description | *Briefly describe the study. For quantitative data include treatment factors and interactions, design structure (e.g. factorial, nested, hierarchical), nature and number of experimental units and replicates.* |
| Research sample | *Describe the research sample (e.g. a group of tagged Passer domesticus, all Stenocereus thurberi within Organ Pipe Cactus National Monument), and provide a rationale for the sample choice. When relevant, describe the organism taxa, source, sex, age range and* |

| | |
|---|---|
| | *any manipulations. State what population the sample is meant to represent when applicable. For studies involving existing datasets, describe the data and its source.* |
| Sampling strategy | *Note the sampling procedure. Describe the statistical methods that were used to predetermine sample size OR if no sample-size calculation was performed, describe how sample sizes were chosen and provide a rationale for why these sample sizes are sufficient.* |
| Data collection | *Describe the data collection procedure, including who recorded the data and how.* |
| Timing and spatial scale | *Indicate the start and stop dates of data collection, noting the frequency and periodicity of sampling and providing a rationale for these choices. If there is a gap between collection periods, state the dates for each sample cohort. Specify the spatial scale from which the data are taken* |
| Data exclusions | *If no data were excluded from the analyses, state so OR if data were excluded, describe the exclusions and the rationale behind them, indicating whether exclusion criteria were pre-established.* |
| Reproducibility | *Describe the measures taken to verify the reproducibility of experimental findings. For each experiment, note whether any attempts to repeat the experiment failed OR state that all attempts to repeat the experiment were successful.* |
| Randomization | *Describe how samples/organisms/participants were allocated into groups. If allocation was not random, describe how covariates were controlled. If this is not relevant to your study, explain why.* |
| Blinding | *Describe the extent of blinding used during data acquisition and analysis. If blinding was not possible, describe why OR explain why blinding was not relevant to your study.* |

Did the study involve field work? ☐ Yes ☒ No

# Reporting for specific materials, systems and methods

We require information from authors about some types of materials, experimental systems and methods used in many studies. Here, indicate whether each material, system or method listed is relevant to your study. If you are not sure if a list item applies to your research, read the appropriate section before selecting a response.

## Materials & experimental systems

| n/a | Involved in the study | |
|---|---|---|
| ☐ | ☒ | Antibodies |
| ☐ | ☒ | Eukaryotic cell lines |
| ☒ | ☐ | Palaeontology and archaeology |
| ☐ | ☒ | Animals and other organisms |
| ☒ | ☐ | Human research participants |
| ☒ | ☐ | Clinical data |
| ☒ | ☐ | Dual use research of concern |

## Methods

| n/a | Involved in the study | |
|---|---|---|
| ☒ | ☐ | ChIP-seq |
| ☒ | ☐ | Flow cytometry |
| ☒ | ☐ | MRI-based neuroimaging |

## Antibodies

| | |
|---|---|
| Antibodies used | ADRA2C antibody (Novus, #NB110-39031, 1:200 for IHC, 1:500 for WB)<br>Ki67 (Abcam, # ab16667, 1:200)<br>FOXO1 (CST, #2880, 1:200 for IHC, 1:1000 for WB)<br>PRL (Abcam, #ab268110, 1:250)<br>IGFBP-1 (Abcam, #ab180948, 1:1000)<br>PKA (CST, # 4782, 1:1000)<br>p-PKA (CST�#9621, 1:1000)<br>ERK (CST�#4695, 1:1000)<br>p-ERK (CST�#8544, 1:1000)<br>AKT (CST, #4691, 1:1000)<br>p-Akt(Thr308) (CST, #13038, 1:1000)<br>p-Akt(Ser473) (CST, #4060, 1:1000)<br>HOXOA10 (Santa Cruz, #sc-271139, 1:1000)<br>STAT3 (CST, #12640, 1:1000)<br>FOXO1(CST, #2880, 1:1000)<br>GAPDH (CST, #5174, 1:2000)<br>H3 (Abmart, # T56587, 1:2000) |
| Validation | Most of the antibodies have been pre-validated by the manufacturer and previously reported in the literature. All antibodies were validated in the lab by band specificity and product size in western blot experiments. Antibodies for ADRA2C was further validated for quantitative detection of decreases in protein expression following knock-down with gene specific siRNA. |

# Eukaryotic cell lines

Policy information about cell lines

| | |
|---|---|
| Cell line source(s) | The immortalized human endometrial stromal cell line purchased from American Type Culture Collection (ATCC) RCRL-4003TM cell. |
| Authentication | As descried in our manuscript materials and methods: The immortalized human endometrial stromal cell line was used for studing the function and mechanism of ADRA2C in vivo in cell proliferation and decidualization status. |
| Mycoplasma contamination | Aliquots of cell culture supernatants from cells in active culture were evaluated regularly for mycoplasma contamination using a PCR-based method. All cell line experiments were performed within 2-3 months of resuscitation of frozen cell stocks prepared with 3 passages of receipt. |
| Commonly misidentified lines (See ICLAC register) | N/A |

# Animals and other organisms

Policy information about studies involving animals; ARRIVE guidelines recommended for reporting animal research

| | |
|---|---|
| Laboratory animals | As mentioned in our materials and methods:" Adult ICR mice (age: 7–8 weeks were purchased from the Vital River Laboratories Co. Ltd. were used in our study." |
| Wild animals | N/A |
| Field-collected samples | As described in our material and methods:" All animal experiments were approved by the animal ethic committee and the Institutional Animal Care. All mice were housed in a 12-h light/12-h dark cycle (light on 07:00 AM-19:00 PM), and free water and food were provided at a temperature of 23°C. Mice were randomly assigned to groups at the time of purchase. No data sets were excluded from the analyses. These animals were examined daily for health issues by qualified personnel; the health status was normal for all animals. The stress model test was performed in female mice pre-stressed through forced swimming in water at 15°C (cold water swimming) for 15 min.The mice were intravenously injected with epinephrine (100 μg/mice) (MedChem Express, Monmouth Junction, USA) in saline at 08:30 AM during days 5-7, once a day, to establish a mouse model of exogenous epinephrine in the early pregnancy period." |
| Ethics oversight | Our animal experiments are conducted in strict accordance with the requirements of the animal ethics committee. |

Note that full information on the approval of the study protocol must also be provided in the manuscript.
